# Combined in vivo muscle mass, muscle protein synthesis and muscle protein breakdown measurement: a ‘Combined Oral Stable Isotope Assessment of Muscle (COSIAM)’ approach

**DOI:** 10.1007/s11357-021-00386-2

**Published:** 2021-05-27

**Authors:** Jessica Cegielski, Daniel J. Wilkinson, Matthew S. Brook, Catherine Boereboom, Bethan E. Phillips, John F. R. Gladman, Kenneth Smith, Philip J. Atherton

**Affiliations:** 1 MRC-Versus Arthritis Centre for Musculoskeletal Ageing Research and NIHR Nottingham BRC, Clinical, Metabolic and Molecular Physiology, School of Medicine, University of Nottingham, Royal Derby Hospital Centre, Uttoxeter Road, Derby, DE22 3DT UK; 2 Nottingham University Hospitals NHS Trust, Nottingham, UK

**Keywords:** Stable isotope tracer, Skeletal muscle, Protein synthesis

## Abstract

Optimising approaches for measuring skeletal muscle mass and turnover that are widely applicable, minimally invasive and cost effective is crucial in furthering research into sarcopenia and cachexia. Traditional approaches for measurement of muscle protein turnover require infusion of expensive, sterile, isotopically labelled tracers which limits the applicability of these approaches in certain populations (e.g. clinical, frail elderly). To concurrently quantify skeletal muscle mass and muscle protein turnover i.e. muscle protein synthesis (MPS) and muscle protein breakdown (MPB), in elderly human volunteers using stable-isotope labelled tracers i.e. Methyl-[D_3_]-creatine (D_3_-Cr), deuterium oxide (D_2_O), and Methyl-[D_3_]-3-methylhistidine (D_3_-3MH), to measure muscle mass, MPS and MPB, respectively. We recruited 10 older males (71 ± 4 y, BMI: 25 ± 4 kg^.^m^2^, mean ± SD) into a 4-day study, with DXA and consumption of D_2_O and D_3_-Cr tracers on day 1. D_3_-3MH was consumed on day 3, 24 h prior to returning to the lab. From urine, saliva and blood samples, and a single muscle biopsy (vastus lateralis), we determined muscle mass, MPS and MPB. D_3_-Cr derived muscle mass was positively correlated to appendicular fat-free mass (AFFM) estimated by DXA (r = 0.69, P = 0.027). Rates of cumulative myofibrillar MPS over 3 days were 0.072%/h (95% CI, 0.064 to 0.081%/h). Whole-body MPB over 6 h was 0.052 (95% CI, 0.038 to 0.067). These rates were similar to previous literature. We demonstrate the potential for D_3_-Cr to be used alongside D_2_O and D_3_-3MH for concurrent measurement of muscle mass, MPS, and MPB using a minimally invasive design, applicable for clinical and frail populations.

## Introduction

Skeletal muscle is the largest organ of the body by mass, accounting for 30–50% of whole-body total protein turnover [1–3]. It contributes to approximately 60% of basal metabolic rate [4]. Loss of muscle mass and function (sarcopenia) is linked to ill-health and clinical outcomes in ageing, and conditions such as diabetes and cachexia [5, 6]. Skeletal muscle metabolism is responsive to anabolic interventions such as exercise, nutrition, and certain pharmaceuticals [7–9]. The three main, related, parameters of muscle metabolism are: muscle mass, muscle protein synthesis (MPS), and muscle protein breakdown (MPB). As such, measurement of all three aspects within a single protocol is desirable.

Muscle volume and, by inference, mass can be measured using DXA, MRI and CT [10]. These techniques are minimally invasive; however, access to them for use can often be limited as they are costly, not readily accessible, and some (i.e. DXA and CT) expose the participant to ionising radiation [11]. DXA is the most ubiquitously employed imaging approach in skeletal muscle research, although it is subject to overestimation of muscle mass [12, 13]. This issue has led to the recent re-emergence of the stable isotope labelled tracer creatine technique [12], using methyl-[D_3_]-creatine (D_3_-Cr) to quantify whole-body skeletal muscle mass, as a minimally invasive, inexpensive, and accurate alternative to the aforementioned imaging approaches. Following ingestion of a known dose of D_3_-Cr, the amount retained in the body can be determined from the quantification of the amount of labelled creatine that ‘spills over’ in a subsequent 24-h pooled urine sample. The ratio of labelled to unlabelled creatinine (which is derived from the conversion of creatine to creatinine in the total muscle pool [14]) in urine samples taken at 48 and 72 h after ingestion of D_3_-Cr is then determined, and from these measurements, the size of the whole body muscle creatine pool can be estimated [15]. A further simple calculation can then estimate total whole-body skeletal muscle mass, assuming that there is 4.3 g creatine per kg of muscle [12]. Using this approach, Clark et al. (2014) established a strong correlation (r = 0.868) with whole-body muscle mass assessement using MRI.

MPS in vivo has traditionally been quantified using measures of uptake of stable isotopically labelled amino acids administered as an intravenous bolus or infusion [16–18], and this approach has been used experimentally to compare a baseline state (such as resting or fasted) with that after an intervention (e.g. feeding, exercise or a pharmacological agent) [9, 19, 20]. However, the quantification of ‘free-living’, cumulative MPS using non-substrate specific deuterium (D_2_O) tracing methods has recently emerged as an important advancement in the field. In these methods, deuterium is rapidly equilibrated in the body water pool (over 1–2 h), exchanges onto amino acids (such as alanine) through intermediary metabolic pathways and is subsequently incorporated into protein [21]. Measurement of these processes permits the quantitation of cumulative MPS over varying time periods from hours or days [22] to weeks or months [9, 19]. As such, these approaches can be applied in free-living people outside of a laboratory, which facilitates their use in the study of, for example, nutrition and physical (in)activity/exercise over clinically meaningful time periods.

The quantification of MPB is arguably more difficult than both muscle mass and MPS. Traditional in vivo approaches include arterio-venous (A-V) balance and fractional breakdown methods [23, 24], quantifying the dilution of amino acid tracers across the A-V system of an isolated organ or limb following the breakdown of tissue protein. These invasive approaches are reliant upon robust and regular blood sampling and/or blood flow measures, both of which have inherent limitations, such as the practicality of numerous and correctly timed blood samples [25]. Another approach has been to measure the concentration of 3-methylhistidine (3-MH) in urine. 3-MH is a post-translational modification of contractile protein histidine residues, which are not subject to re-incorporation into nascent peptide synthesis, as there is no aminoacyl-tRNA for 3-MH [26]. However, this approach lost favour as urinary 3-MH levels may be perturbed by dietary meat intake [27] (an alternative source of 3-MH); and the assumption that methylated-histidine is exclusively derived from skeletal muscle of the appendicular skeleton may be incorrect, as the gut has also been shown to be a source of 3-MH [28]. Nevertheless, an adaptation of this approach using tracer dilution was reported recently in which 10 mg of stable isotopically labelled 3-methylhistidine (either D_3_ or ^13^C) was administerd orally, and the rate of dilution of this tracer by endogenously released 3-MH was determined in plasma or urine over 6 h to assess the rate of whole-body myofibrillar protein breakdown [26]. This method offers a simple, attractive alternative which does not require accurate and complete 24-h urinary collection because a much more easily collected series of spot urine or plasma samples can be used.

To date, there has been no attempt to create a single protocol to streamline minimally invasive techniques to simultaneously measure muscle protein turnover and muscle mass. Being able to do so would have considerable clinical research application in non-laboratory settings (i.e. in the community, hospitals or care homes) and/or in patient groups who find it difficult to comply with, or understand, invasive approaches, such as frail individuals or those with disabilities. Therefore, the aim of this study was to develop a single combined oral stable isotope assessment of muscle (which we term ‘COSIAM’) to concurrently quantify skeletal muscle mass and muscle protein turnover; MPS and MPB, using stable isotope methods with D_3_-Cr, D_2_O and D_3_-3MH, respectively.

## Methods

### Study protocol

This study was approved by The University of Nottingham Ethics Committee (A08122015) and conformed to the standards set by The Declaration of Helsinki (2013).

Ten healthy older males were recruited (71 ± 4y, BMI: 25 ± 4 kg^.^m^2^, mean ± SD). Before being enrolled into the study, all participants had a full medical screening. Height, weight, full medical history, blood pressure, an ECG and clinical chemistry (full blood count, urea and electrolytes, liver function tests, thyroid function tests and coagulation) were used to assess subject eligibility to participate in the study. Participants were excluded if they had a BMI > 35 kg·m^2^, active cardiovascular disease, cerebrovascular disease, respiratory disease, metabolic disease, inflammatory bowel or renal disease, active malignancy, any musculoskeletal or neurological disorders, any signs of clotting dysfunction, any recent steroid treatment (within 6 months), or if on hormone replacement therapy. All participants were informed of the purpose of the study and of all the risks and procedures involved, before providing their written, informed consent.

On the first study day (Day 1), participants were asked to attend the unit at 8:30 am for a whole-body DXA scan, having fasted overnight. They provided a baseline saliva sample (to identify baseline body water enrichment) and then performed the Short Physical Performance Battery Test (SPPBT). Leg extensor strength (single-leg 1RM and maximal voluntary contraction for leg extension) and handgrip strength (using Takei (T.K.K. 5401 GRIP-D) handgrip dynamometer) were also measured. A baseline 10 ml blood sample was taken to measure background blood/plasma alanine labelling prior to D_2_O exposure. A baseline urine sample was collected to measure D_3_-creatinine enrichment. Participants were then given three 50 ml aliquots of 70 atom percent D_2_O to drink, each taken 25 min apart to minimise the risk of side effects such as dizziness or nausea that are sometimes reported after D_2_O consumption due to disturbance of specific gravity within the vestibular fluid [29]. 30 mg D_3_-Cr was included in the final 50 ml aliquot of D_2_O.

Two hours after consumption of the final D_2_O aliquot, participants provided another saliva sample, to be used to determine the plateau D_2_O concentration in body water.

**Fig. 1:**
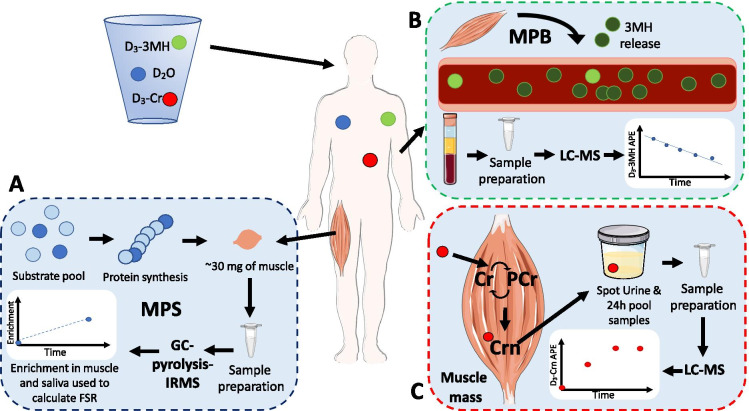
Schematic representation of the combined use of D_3_-3MH, D_2_O and D_3_-Cr to measure myofibrillar muscle protein synthesis (MPS) (**A**), whole-body muscle protein breakdown (MPB) (**B**), and muscle mass (**C**), simultaneously

Prior to leaving the laboratory, participants were provided with a container to collect all urine for 24 h, a tube for a spot urine sample at 48 h and three tubes for saliva samples at 12, 24 and 48 h after the final D_2_O aliquot was consumed. These samples were used to measure D_3_-creatine spill-over, urinary D_3_-creatinine enrichment and the size of the creatine pool, respectively. Participants were also given 10 mg of D_3_-3MH dissolved in 50 mL of water to take home and store refrigerated. Participants were instructed to consume the D_3_-3MH immediately after the 48-h samples were collected (Fig. 1). Please refer to Fig. 1 for an illustrative depiction of the COSIAM approach.

**Fig. 2:**
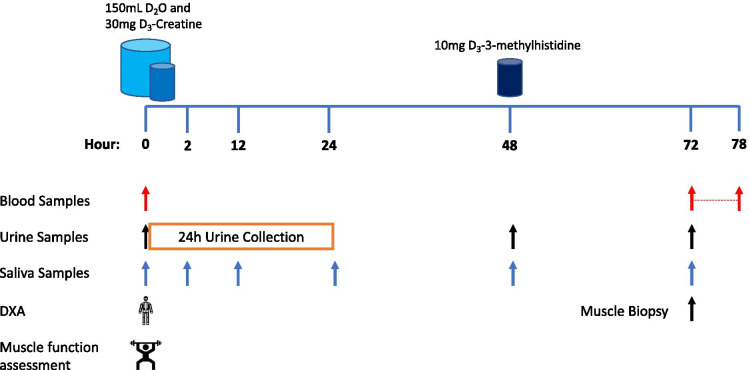
Study schematic

Participants returned to the laboratory 72 h after consumption of the final D_2_O aliquot given on the first study visit, again having fasted overnight. At this visit, hourly bloods were collected over 6 h (between 72 and 78 h after consumption of the final D_2_O aliquot), plus a further spot urine and saliva sample at 72 h. A muscle biopsy from the *m. vastus lateralis* (VL) was also taken at this visit (at 72 h) under local anaesthetic (1% lidocaine). The Bard micro-needle biopsy technique (50% length of VL, mid-belly) was used for the first 5 participants, with the conchotome approach [30] used subsequently to achieve a larger tissue yield for further analyses. Participants were not fasted throughout the blood sampling period, with a standardised light meal (sandwich, crisps, yoghurt and drink) provided at the midpoint of the blood collections for all participants. Figure 2 illustrates the protocol.

### Sample analysis

#### Measurement of body water enrichment in saliva, for the estimation of alanine precursor enrichment

One hundred microlitres of each saliva was aliquoted into the cap of auto-sampler vials which were crimped onto inverted vials and placed on a heating block at 90 °C for 4 h. These vials were then immediately cooled upright on ice for 10 min and the water collected transferred to fresh vials. The samples were then injected into a high temperature conversion elemental analyser (TC/EA; Thermo Scientific, Hemel Hempstead, UK) connected to an Isotope Ratio Mass Spectrometer (IRMS; Delta V Advantage, Thermo Scientific) [20]. The mean of the final 3 of 4 repeat injections was taken to control for carry over from previous samples. The mean area under the curve (AUC) of the atom percent excess (APE) was used to represent the average precursor labelling of free intramuscular alanine, multiplied by 3.7 to account for deuterium exchange into alanine from D_2_O, for the determination of muscle fractional synthetic rate (FSR).

#### Preparation of muscle for measurement of incorporation of deuterated alanine, for MPS determination

Between 30 and 50 mg of muscle was used for the analysis of deuterium labelling of alanine in the myofibrillar protein bound fraction, although 10 mg is sufficient, thereby permitting the needle biopsy approach. The muscle tissue was homogenised with scissors in an Eppendorf tube using 10 μL/mg^−1^ of ice-cold homogenisation buffer (50 mmol Tris–HCl, 1 mmol EDTA, 1 mmol EGTA, 10 mmol β-Glycerophosphate, 50 mmol NaF, 0.5 mmol of activated sodium orthovanadate, pH 7.4 (all from Sigma Aldrich, Poole, UK) containing a complete protease inhibitor cocktail tablet (Roche, West Sussex, UK). The homogenate was mixed at 1000 rpm for 10 min before being centrifuged at 10,000 × g for 10 min at 4 °C. The supernatant (sarcoplasmic fraction) was then collected and the pellet was resuspended in 500 μL mitochondrial extraction buffer (MEB) and then homogenized by Dounce and centrifuged at 1000 g for 5 min. The myofibrillar pellet was solubilised using 1 mL 0.3 molL^−1^ NaOH and incubated for 30 min at 37 °C, then centrifuged for 10 min, at 4 °C and 10,000 rpm. The myofibrillar fraction (supernatant) was separated from the insoluble collagen pellet, and precipitated by the addition of 1 mL 1 molL^−1^ perchloric acid, following incubation at 4 °C for 20 min. The myofibrillar protein was pelleted at 4000 rpm for 20 min, separated from the supernatant containing the free amino acids, then washed twice with 70% ethanol. After the addition of 1 mL 0.1 molL^−1^ HCl and 1 mL of a Dowex H^+^ resin slurry, the samples were incubated overnight at 110 °C in order to release the protein-bound amino acids [20, 31, 32]. The plasma was deproteinised using ice-cold ethanol (100%) and centrifuged at 17,000 g for 10 min. The pellet was transferred to a boiling tube containing on dowex with 0.1 M HCl and hydrolysed, overnight at 110 °C 16–20 h; following the same preparation steps as the muscle sample.

The amino acids (AA) were derivatized as their N-methoxycarbonyl methyl esters (MCME) as previously described [33]. Briefly, the AA were re-suspended in 60 μL of distilled H_2_O and vortex mixed before the addition of 32 μL of methanol and 10 μL of pyridine. 8 μL of methylchloroformate (MCF) was carefully added directly into the aqueous mix and immediately vortex mixed for 30 s. The solution was left at room temperature for 5 min to react. Following the addition of 100 μL of chloroform and 100 μL of 0.001 M NaHCO_3_, the samples were vortexed mixed to extract the MCME amino acids. Molecular sieve was added to remove any excess water from the sample. The sample was transferred to an autosampler vial, ready for mass spectrometric analysis. Deuterium enrichment into protein-bound alanine was measured using gas-chromatography-pyrolysis-isotope-ratio-mass spectrometry (GC-pyrolysis-IRMS, Delta V Advantage, Thermo Scientific) [34].

The calculation of myofibrillar fractional synthetic rate was based on the following product-precursor equation:

$$\mathrm{FSR} \left(\%/\mathrm{h}\right)=-\mathrm{In}\left[\frac{1-\left(\frac{{APE}_{Ala}}{{APE}_{p}}\right)}{t}\right]$$

where APE_Ala_ is deuterium enrichment of protein-bound alanine, APE_P_ is mean alanine enrichment corrected from D_2_O, and t is the time between the plasma sample to the 72 h muscle biopsy, using previously described equations [19].

#### *Measurement of D*_*3*_*-3-methyl-histidine in blood, for MPB determination*

Blood was taken, then centrifuged at (3200 rpm, 20 min, 4 °C), the plasma aliquoted into eppendorfs and stored at − 80 °C. Plasma samples were defrosted and centrifuged at ~ 10,000 rpm for 3 min. A 0–10% D_3_-3-methyl-histidine enrichment curve was prepared as a serial dilution. 100 µL of plasma was de-proteinised using 1 mL of MeCN: MeOH (1:1). Samples were vortex mixed and incubated at − 20 °C for 1 h. Samples were centrifuged at ~ 13,000 rpm for 5 min at 4 °C. The supernatant was dried down in a Techne Block at < 40 °C using nitrogen gas. Samples were re-suspended using 100 µL MeCN: ddH_2_O (65:35) and ready to be analysed using High-Performance Liquid Chromatography (HPLC; Dionex Ultimate 3000, Thermo Scientific) mass spectrometry (MS; Q-Exactive, Thermo Scientific) with a Sequant ZIC-HILIC column (150 mm × 2.1 × 5um; Merck Millipore). The flow was set to 0.4 mL/min with an initial buffer gradient of 95:5 (Buffer A:B); where Buffer A was 10 mM ammonium formate (90:10 Acetonitrile: ddH_2_O) with 0.1% Formic Acid and Buffer B was 10 mM ammonium formate (50:50 Acetonitrile: ddH_2_O) with 0.1% Formic acid. After a 2.5-min hold at 95:5 (A:B), the buffer gradient was ramped to 100% Buffer B over 15 min and held for 2.5 min before returning back to 95:5 (A:B) and re-equilibrated for 10 min. Accurate mass single ion monitoring was performed for M^+^H (mass + hydrogen cation): 170.09230 (3MH) and 173.11131 (D_3_-3MH) to determine enrichment of D_3_-3MH. The enrichment ratios were log transformed to determine the decay rates (k), representative of the rate of whole-body MPB [26].

#### Determination of creatine spillover and creatinine enrichment in urine samples for calculation of whole body muscle mass

The urine samples were prepared using a method based on that described by Leonard et al. [35]. Samples were freeze-thawed (1 ×) at room temperature and vortex mixed, before 10 µL of ^13^C-Creatine (20 µg/mL) was added to 50 µL of urine, as an internal standard. 250 µL of ice-cold acetonitrile was added both samples and standards, vortex mixed and cooled on ice for 30 min. Samples were then centrifuged at 17,000 g for 20 min. The supernatant was filtered through a 0.2 µm filter and transferred to vials ready for HPLC–MS analysis using the same instrumentation and column as above [35]. The flow was set to 0.2 mL/min, 60:40 (Buffer A:B) isocratic flow; where Buffer A was 100% Acetonitrile and Buffer B Ammonium Acetate pH 5.8. A standard curve using ^12^C, ^13^C and D_3_-Creatine was prepared for the determination of creatine concentration and enrichment (monitoring M + H: 132.07666, 133.08006 and 135.09549 respectively), with a D_3_-creatinine enrichment curve of 0–0.1% for determination of D_3_ − creatinine enrichment (monitoring M + H: 114.06638 and 117.08515). From the measurement of urinary D_3_ − creatine in the 24-h urine (spillover) and D_3_ − creatinine enrichment in the 48 h and 72 h spot urines, total creatine pool size and therefore total whole body muscle could be calculated using the following equation:

$$\mathrm{Total}\ \mathrm{muscle}\ \mathrm{mass}=\frac{\left(\frac{MW_{Unlabelled}}{MW_{labelled}}\right)\times \left( Amount\ of\kern0.5em {D}_3- Cr\kern0.5em dosed\kern0.5em (g)- Amount\ of\ {D}_3- Cr\kern0.5em excreted\kern0.5em (g)\right)}{\left( mean\kern0.5em steady- state\kern0.5em {D}_3- Cr eatinine\kern0.5em enrichment\kern0.5em ratio\right)\kern0.5em }\div 4.3\left(g/ kg\right)$$

where ${MW}_{Unlabelled}$ and ${MW}_{labelled}$ represent the molecular weights of both unlabelled (131.1) and labelled creatine (134.1), respectively. The estimated creatine pool size is then divided by 4.3 g/kg which reflects the average concentration of creatine found in whole wet muscle tissue [12].

### Statistical analyses

As the methods have individually been previously validated, here we aimed to measure rates of MPS and MPB using our COSIAM approach, to identify if values obtained were similar to previous literature. Furthermore, we aimed to confirm if there was a relationship between DXA and D_3_-creatine-derived estimates of muscle mass. All data sets were tested for normality using Shapiro–Wilk test with alpha set at 0.05. Linear regression and two-tailed Pearson r correlation analysis were used to identify any relationship between muscle mass estimates by D_3_-creatine and DXA, with confidence interval set at 95%. All statistical analysis was performed using Graphpad Prism software (Prism 8, USA).

## Results

### Myofibrillar protein synthesis and body water enrichment

**Fig. 3:**
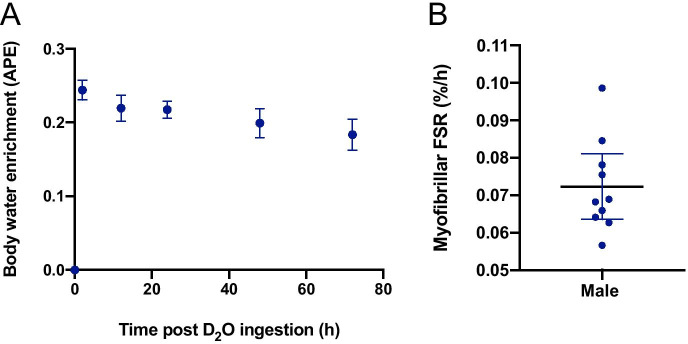
**A** Deuterium enrichment of the body water, measured in APE from saliva samples at baseline, 2, 12, 24, 48 and 72 h post-deuterium oxide ingestion; (**B**) Myofibrillar fractional synthetic rates. n = 10 older males. Mean and 95% CI presented

The mean body water enrichment of D_2_O peaked at 2 h (0.24 APE; 95% CI, 0.23 to 0.26 APE) and then declined slowly over 72 h to 0.18 APE (95% CI, 0.16 to 0.20 APE; Fig. 3A). The mean myofibrillar FSR over 0–72 h was 0.072%/h (95% CI, 0.064 to 0.081%/h; Fig. 3B).

### *Determination of rates of muscle protein breakdown (MPB) by D*_*3*_*-3-methylhistidine dilution*

**Fig. 4:**
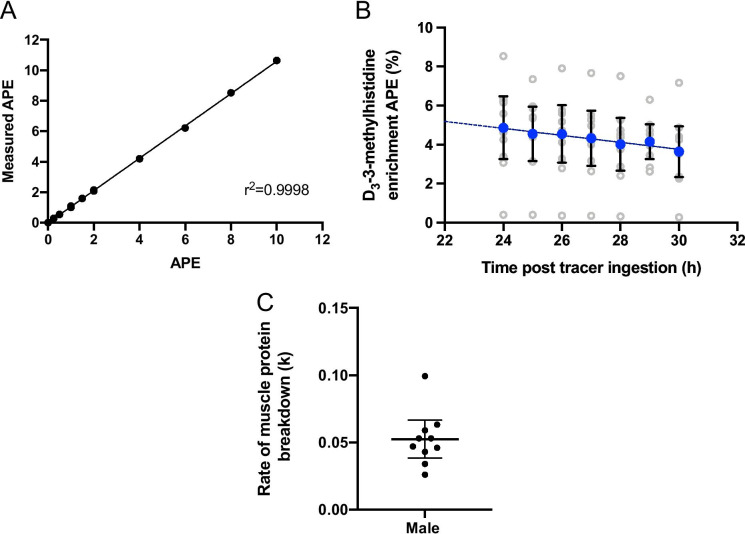
**A** Enrichment curve of D_3_-3-methylhistidine from 0–10 APE; (**B**) Decay curves of D_3_-3-methylhistidine in the plasma by endogenous 3-methylhistidine release; (**C**) Rate of whole-body muscle protein breakdown (k) using D_3_-3-methylhistidine. n = 10 older males. Mean and 95% CI presented

The D_3_-3MH enrichment curve of 0–10 APE (Fig. 4A) showed good linearity, whereby APE measured at different enrichments were identical to the expected APE (slope = 1.060). Mean plasma D_3_-3-MH enrichment gradually declined from 4.86 ± 2.25 APE at 24 h post-tracer ingestion to 3.65 ± 1.82 APE at 30 h (Fig. 4B). The ratio of labelled to unlabelled 3-MH was log transformed to determine the rate of whole-body MPB. The gradient of the straight line provides the k value (rate constant). The mean rate of whole-body MPB was 0.052 (95% CI, 0.038 to 0.067; Fig. 4C).

### *Determination of muscle mass using D*_*3*_*-Creatine*

**Fig. 5:**
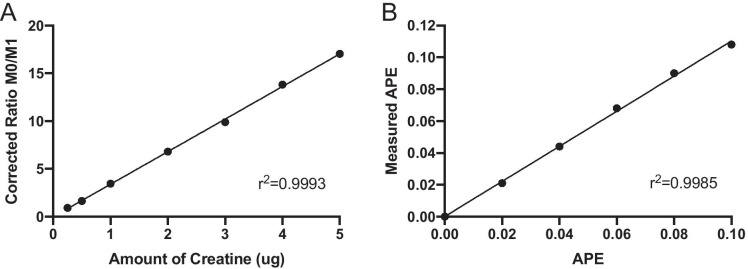
**A** Creatine concentration curve ranging from 0.25–5 µg of creatine where M0 is creatine and M1 is ^13^C-creatine; (**B**) D_3_-creatinine enrichment curve ranging from 0 to 0.1 APE for measured vs. expected APE

**Fig. 6:**
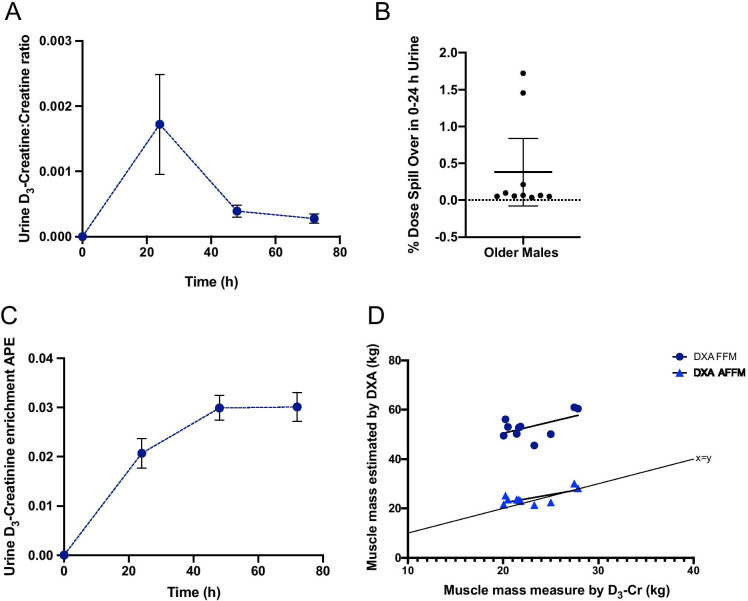
**A** Corrected ratio of D_3_-Creatine/Creatine excreted in the urine for samples collected at Baseline (0 h), 24 h (pool), 48 and 72 h (spot); (**B**) Percentage dose not absorbed by the body in 0–24 h urine collection sample. Values displayed are min to max; (**C**) Ratio of labelled creatinine (D_3_-creatinine) to unlabelled creatinine, corrected to the baseline sample (0 h) for 24, 48 and 72 h urine samples. Values displayed are mean ± 95% CI; (**D**) Correlation between muscle mass measured by D_3_-Creatine method with fat free mass (FFM) and appendicular fat free mass (AFFM i.e. total arms and legs) measured by DXA. Values displayed are mean ± 95% CI

This analytical approach was extremely sensitive, accurate and robust, and both standard curves showed good linearity across both the range of concentrations of creatine expected (i.e. 0.25 to 5 µg; Fig. 5A) and expected D_3_-Cr enrichments (i.e. 0–0.1APE; Fig. 5B). In our hands, only very small amounts of D_3_-Cr were excreted during the 0–24 h collection period (0.38% of the initial dose; Fig. 6B). D_3_-Cr enrichment in the urine samples increased over time and plateaued at 48 h, there was no significant difference between 48 and 72 h enrichments (Fig. 6C); as such, the mean enrichment values of the 48 h and 72 h urine samples were then used to calculate muscle mass. There was no significant correlation between DXA-derived FFM and muscle mass measured by D_3_-Cr (r = 0.55, p = 0.098). However, DXA-derived AFFM was significantly positively correlated with muscle mass measured by D_3_-Cr (r = 0.69, p = 0.027; Fig. 6D).

## Discussion

We present a minimally invasive study designed to measure rates of MPS, MPB and muscle mass concurrently, using a novel combination of orally ingested stable isotope labelled tracers, and mass spectrometric measures made in saliva, and blood or urine samples, in conjunction with a single muscle biopsy. In contrast to traditional stable isotope approaches using intravenous tracer infusions, which require extremely well-controlled laboratory conditions and imaging techniques, our protocol has the potential to be applied to a wider range of cohorts and settings.

In our protocol, we used a creatine tracer method to quantify muscle mass. The use of 30 mg D_3_-Cr tracer was based upon the dosing validation by Clark and colleagues in 2014 where 30 mg was identified as adequate for quantification of muscle mass, a finding that we support. Our results also corroborate previous relationships between DXA-derived parameters of skeletal muscle and muscle mass measured using D_3_-creatine [12, 36, 37]. A significant correlation was only found between muscle mass measured by D_3_-creatine and AFFM calculated from DXA (and not with FFM by DXA), this may be due to the fact that DXA does not measure muscle mass directly. Our findings are supported by those of Proctor et al., who found the strength of relationship between a urinary creatinine excretion method and DXA for assessing FFM declined with age [38]. In addition, FFM as measured by DXA is considerably higher (~ 60%) than the muscle mass determined using D_3_-creatine. Therefore, if DXA FFM is used to determine muscle mass, it will provide an erroneous overestimation of total muscle [and by extenstion, contractile] mass [39]. Ideally, the gold-standard approach to determine muscle mass (i.e., MRI) would have been used for comparison, however, a very close correlation between MRI and D_3_-creatine has been previously documented [12]. Although there is a strong correlation between AFFM using DXA or MRI, and muscle mass measured by D_3_-creatine, a potential limitation that requires further validation, is the widespread use of the 4.3 g creatine per kg muscle constant to calculate muscle mass based upon the size of the creatine pool [12]. It is important to ensure that the constant used accurately reflects intramuscular creatine in different populations, particularly where the muscle creatine pool size may vary e.g. vegetarians [40], athletes, especially following acute or endurance exercise [41] or when creatine is being supplemented in the diet [42]. When considering the impact of dietary intake, although a large amount of creatine can be found in meat, average ingestion is ~ 1 g/day [43] in an omnivorous diet. In an average 70 kg young male the creatine pool size is approximately 120–140 g [43] with creatine excretion approximately 1.7%/day [44] as such average meat ingestion should not affect this measure; indeed significant changes in creatine pool size (~ 20%) have only been observed when supplementating creatine of 20 g/day over 6 days [45].

Given the precision of the D_3_-Cr measurement in our hands, we calculate it would be possible to detect a change in muscle mass of 1 kg, equal to ~ 0.03 APE, and therefore has the sensitivity to identify clinically meaningful changes [46]. Future applications of this approach could also look to minimise the urine sampling to a single spot urine, given that the spillover is less than 0.4% in our participants, perhaps due to the lower dose given, and this would lead to < 0.4% error in muscle mass calculation. We speculate that the percentage spillover may be related to the creatine pool size and thus, muscle mass and should be investigated further. Going forward, we suggest that 1 or 2 spot urine samples taken 48–72 h after ingestion of tracer would suffice, making the method even more practicable and widely applicable. To exemplify, in this data set, removing the correction of the 24 h collection, led to an average increase in muscle mass estimation of 9 g. Alternatively, Shankaran et al. (2018), developed an algorithm to correct for D_3_-Cr spillage based on a relationship identified between fasting creatine:creatinine ratio and D_3_-Cr dose excreted from a urine sample, thereby, removing the need for the 24 h pooled collection [47].

Using D_2_O to study skeletal muscle protein metabolism is now a well-established stable-isotope tracer technique that has been validated for both longer (over weeks or months; [19]) and also relatively short study durations i.e. over 2–8 days [22], and even over as little as 3 h with appropriate increases in the pre-loading dose [20]. For instance, as little as 150 mL (70 AP) D_2_O) was adequate to measure MPS over 2–8 days [22]; therefore, we used this dose to measure MPS in this 4-day protocol. In doing so, we report similar cumulative MPS values to those reported before [22, 48] and interference with the other co-consumed tracers D_3_-Cr or D_3_-3MH (i.e. D_2_O contaminating creatine/methylhistidine labelling) is negligble. It is unlikely that a deuterium labelling of either arginine or glycine (amino acids involved in the initial step of creatine biosynthesis) from D_2_O would be incorporated to any extent into D_3_-labelled guanidinoacetate (a precursor to creatine), given the very small dose of D_2_O we use in this study, and therefore the probability of D_2_O labelling all three position of the methyl group at this level of dosing is vanishingly small. In addition, it is possible to provide an alternatively labelled tracer such as ^13^C-Cr for repeat measurements, and this would be recommended in short duration longitudinal studies to avoid any overlap of isotopes given the slow turnover of these pools [22].

For measurement of whole-body MPB, 10 mg of D_3_-3MH was utilised, as previously described by Sheffield-Moore and colleagues, with the rates of MPB in our older men similar to those previously observed (i.e. the rate constant (k) of approximately 0.08, [26]). Sheffield-Moore et al. (2014), reported no significant difference between the use of meat or meatless diets in their study and therefore diet was not controlled for in our study. It is important to note that subjects were not maintained fasted throughout the period of plasma sampling for D_3_-3MH measures, therefore the rates of MPB we observed reflect the changes in MPB over the period of sampling. Feeding increases insulin secretion which is known to inhibit MPB [49]; therefore, it is likely that our measures will somewhat reflect MPB in the fed state, thereby explaining why our MPB rates are slightly lower than the cummulative rates of MPS obtained in this study. One subject had a much lower D_3_-3MH enrichment than the other subjects, which is probably indicative that they did not ingest the full 10 mg or perhaps took the dose much earlier than reported i.e. not the morning before; however, the decay rate determined was similar to that of our other participants, due to the high sensitivity and reproducibility of the LC–MS approach employed. Further investigations could look to reduce the dose of D_3_-3MH given or standardise the dose on a weight or LBM basis to standardise the approach and measurement of enrichment across participants. Furthermore, using timed urine samples, instead of blood samples for the measurement of labelled D_3_-3MH excretion could further minimise the invasiveness of this approach [26].

Other considerations to the work presented herein are warranted. With regard to selecting which timepoint to take the spot samples, Clark et al. (2014) reported that a steady-state of D_3_-creatinine enrichment was achieved by 30.7 ± 11.2 h post-tracer ingestion and remained steady for a further 5 days [12]. As such, a single spot urine sample taken at any timepoint during this steady-state period (2–4 days post-ingestion of tracer) would be sufficient. For more acute measures, the timepoint for MPS measurement could be altered i.e. shortened, and with an additional biopsy to measure MPS, with appropriate sampling over the same time frame (i.e. 6 h) as MPB [20], also permitting us to adopt this approach pre- and post- longer term interventions. Although this would require additional biopsies, the use of the micro-needle biopsy technique [50], which is well tolerated [50], may be used towards developing a less invasive approach. It would also be possible to further shorten the time-frame and burden of these measures by providing all three tracers together on Day 0, obtaining plasma/urine samples the next day for MPB and a muscle biopsy at 24 h for MPS, and a spot urine at 48 h or 72 h post-tracer consumption for muscle mass estimation. Additionally, future adaptations to the COSIAM approach may include the replacement of the muscle biopsy for the measurement of MPS with the use of the “virtual biopsy” approach to measure deuterium labelled plasma surrogates of MPS such as creatine kinase M-type (CK-M) or carbonic anhydrase 3 [51]. While this would further reduce the invasiveness of the approach and should be investigated further, the higher dose of deuterium oxide required for this approach may have a greater burden with the potential of increased side effects e.g. nausea. Furthermore, the relationship of plasma surrogates such as CK-M would require validation across different populations and scenarios. Finally, we speculate that where there is rapid muscle catabolism, such as in sepsis/burns, this approach would be equally valid for quantifying such gross perturbations in proteostasis (in fact one may speculate larger effect sizes would be easier to detect). Nonetheless, oral tracing of D_3_-3MH/D_3_- Cr does require gut/renal function and thus would need to be validated in relation to mapping the fate of oral tracers in populations of potential concern.

This study has demonstrated the feasibility of concurrent assessment of muscle protein turnover (i.e. MPS and MPB), and muscle mass through a far less invasive approach than using traditional stable isotope infusion approaches. The COSIAM approach provides rates of protein turnover and estimates of muscle mass that are in agreement with previous individual tracer approaches. The combination of these tracers via a minimally invasive approach has wider applicability than traditional methods and potential utility to provide novel, valuable information about the regulation of muscle mass in difficult to study clinical populations.
